# Questions and Answers on Dental Subjects

**Published:** 1891-11

**Authors:** Gustavus North


### Questions and Answers on Dental Pathology and Therapeutics, Dental Embryology, Hygiene and Care of Children's Teeth.

BY GUSTAVUS NORTH, A.M., D.D.S.

In the preface the author says, "This book will include the outlines of the questions and answers of about five lectures delivered by the author, on Dental Pathology and Therapeutics, Dental Embryology, Hygiene and the Care of Children's Teeth. This work has been prepared especially for students, and the questions and answers are plain and to the point."

This work is divided into three parts, with questions and answers pertinent and directly to the point. They are so arranged as to carry the student along, upon each subject, in a very efficient and profitable manner. It is quite elementary, as it should be, upon the subjects and for the purpose for which it is designed. Many practitioners can consult it with profit, but its greatest value is to the student who is beginning the study of these branches. The subject of Temperaments is well analyzed, and really constitutes the Fourth Division of the Work. We can most heartily recommend this little work to every Dental student, as one that will be to him many times the value of its cost. It can be obtained of the author, whose address is Springfield, Iowa.

"Three thousand Questions on Medical Subjects," arranged for self-examination with the proper references to standard works, in which the correct replies will be found. Published by P. Blakiston, Son & Co., Philadelphia, Pa.

This house is doing a great service for the medical profession, and for the dental as well, by the issue of quiz compends and such books as the one here indicated. The following from the Publisher's Preface will give a good idea of the scope and value of this little book. "It has been prepared by a medical man, a writer and teacher of experience, with special reference to the actual wants of the medical student. By its help the student can successfully quiz himself on all important branches, or review any one subject in which he feels himself to be particularly deficient. As a rule, the questions have been selected with regard to their bearing upon the practice of medicine and are those most likely to be asked in the quiz class. At the same time, there are many unusual ones, thus giving the student a wide range of thought and making him generally conversant with all points connected with the matter in hand." Unlike many other quiz lists or question books, this makes reference to some standard text-book for the answer to every question. These text-books are the leading ones and such as every medical and dental student must have in their course. The following subjects are embraced in this work, viz : Anatomy, Physiology, Materia Medica, Chemistry, Practice of Medicine, Surgery, Obstetrics, Gynecology, Diseases of Children. These three thousand questions are contained on one hundred and forty-four pages, in small pocket form and interleaved throughout for notes or references. And we may say here confidentially to students of medical science or practice in any department, that the publisher will send this book free upon the student's sending ten cents in postage stamps to cover cost of mailing—certainly a very liberal proposition.

PREPARATIONS for the "*World's Columbian Exposition*" are being pushed forward with astonishing energy and rapidity. Thousands of men are engaged, and structures spring up as if by magic. When completed it will excell anything of the kind the world has ever seen, and notwithstanding this, so great is the interest of all civilized nations, that the present indications are that the demand for space and accommodation, will far exceed the supply. *More anon.*

